# Supercapacitor Cell Performance with Bacterial Nanocellulose and Bacterial Nanocellulose/Polybenzimidazole Impregnated Membranes as Separator

**DOI:** 10.3390/membranes15010012

**Published:** 2025-01-08

**Authors:** Hristo Penchev, Galia Ivanova, Venelin Hubenov, Ivanka Boyadzieva, Desislava Budurova, Filip Ublekov, Adriana Gigova, Antonia Stoyanova

**Affiliations:** 1 Institute of Polymers, Bulgarian Academy of Sciences, “Acad. G. Bonchev” St., Bl.103A, 1113 Sofia, Bulgaria; dbudurova@gmail.com (D.B.); ublekovphilip@gmail.com (F.U.); 2 Institute of Electrochemistry and Energy Systems, Bulgarian Academy of Sciences, G. Bonchev Str. 10, 1113 Sofia, Bulgaria; galia.ivanova@iees.bas.bg (G.I.); a.gigova@iees.bas.bg (A.G.); 3 The Stephan Angeloff Institute of Microbiology, Bulgarian Academy of Sciences, “Acad. G. Bonchev” St., Bl.26, 1113 Sofia, Bulgaria; vhubenov@microbio.bas.bg (V.H.); petrovaim@abv.bg (I.B.)

**Keywords:** bacterial nanocellulose, potassium hydroxide, polybenzimidazole, impregnation, Viledon^®^, symmetric supercapacitor

## Abstract

Supercapacitors are advanced energy storage devices renowned for their rapid energy delivery and long operational lifespan, making them indispensable across various industries. Their relevance has grown in recent years due to the adoption of environmentally friendly materials. One such material is bacterial nanocellulose (BNC), produced entirely from microbial sources, offering sustainability and a bioprocess-driven synthesis. In this study, BNC was synthesized using a symbiotic microbial community. After production and purification, pristine BNC membranes, with an average thickness of 80 microns, were impregnated with an alkali-alcohol meta-polybenzimidazole (PBI) solution. This process yielded hybrid BNC/PBI membranes with improved ion-transport properties. The BNC membranes were then doped with a 6 M KOH solution, to enhance OH^−^ conductivity, and characterized using optical microscopy, ATR FT-IR, XRD, CVT, BET analysis, and impedance spectroscopy. Both BNC and BNC/PBI membranes were tested as separators in laboratory-scale symmetric supercapacitor cells, with performance compared to a commercial Viledon^®^ separator. The supercapacitors employing BNC membranes exhibited high specific capacitance and excellent cycling stability, retaining performance over 10,000 charge/discharge cycles. These findings underscore the potential of BNC/KOH membranes for next-generation supercapacitor applications.

## 1. Introduction

In recent years, the demand for efficient energy storage devices has surged, driven by the finite nature of petroleum resources and the increasing global reliance on them. This trend has spurred a shift towards eco-friendly, renewable energy solutions, necessitating devices with both high energy and power density. Electrochemical capacitors, commonly known as supercapacitors, have gained attention due to their diverse practical applications [1]. Compared to traditional batteries, supercapacitors offer several advantages: a significantly longer lifespan, excellent cycling stability, high power density, low cost, lightweight design, easy portability, and rapid charge–discharge cycles [2,3]. Moreover, the development of supercapacitors from environmentally friendly materials presents a promising area of research. Alkali metal hybrid ion capacitors (AHICs) combine the advantages of batteries and supercapacitors and balance the disadvantages of both devices, which allows high energy and power densities and long cycling life to be maintained simultaneously [4].

Cellulose-based materials are emerging as cost-effective and sustainable alternatives to traditional materials like aluminum, ferrous metals, and rare elements such as ruthenium, gallium, and indium for energy material fabrication [5,6]. Among these, nanocellulose (NC)—the nanoscale derivative of cellulose—stands out due to its remarkable physical, chemical, and thermal properties, which make it suitable for applications in energy storage, sensors, medicine, composites, and hybrid systems [7,8,9]. NC exhibits exceptional mechanical properties, biocompatibility, high specific surface area, crystallinity, purity, amphiphilic nature, surface chemical reactivity, and barrier properties [10]. These characteristics enhance its performance in energy storage devices. Furthermore, its high carbon content and ease of surface modification make NC an excellent raw material for fabricating carbon-based electrodes with a significant surface area. The material’s high thermal and structural stability, combined with its wettability in various electrolytes and broad potential window, positions it as a strong candidate for use in electrochemical energy storage systems [11]. Given these properties, NC and its derived materials have attracted intense research interest in the field of supercapacitors. The ultimate goal is to identify stable, cost-effective, safe, and efficient materials that meet the demands of modern energy storage technologies.

Nanocellulose (NC) plays a versatile role in supercapacitor development, with applications in electrode preparation, electrolyte formulation, and as a separator material. Its utility in electrodes is threefold: it can act as an electrode binder, serve as a structural substrate, or function as a precursor for carbon-based nanomaterials [12]. While NC is inherently non-conductive, its application necessitates combining it with conductive materials such as conductive polymers, carbon-based materials, or metal nanoparticles.

NC-based composite electrodes are widely utilized in supercapacitors. Conductive materials integrated into NC and wood composite electrodes include carbon blacks, carbon nanotubes (CNTs), graphene, metal particles, and conductive polymers [13,14]. These combinations leverage the mechanical robustness and eco-friendliness of NC while enhancing electrical conductivity. For instance, metal particles, with conductivity around 10^5^ S/cm, significantly outperform graphite fibers (~2.2 × 10^4^ S cm^−1^) in conductivity, making them ideal partners for NC in hybrid electrode designs. The inclusion of NC in electrode fabrication also contributes to cost reduction, improved stability, and enhanced electrochemical performance [5].

In addition to its role in electrodes, NC is crucial in the development of polymer-based electrolytes. Incorporating NC into polymer matrices not only reinforces mechanical properties but also promotes enhanced ionic conductivity, making it a valuable additive for high-performance supercapacitor systems [15].

Polymeric and ceramic-based separators are critical components in electrochemical devices, as they prevent device failure by maintaining electrical insulation between the cathode and anode. Nanocellulose (NC) offers a promising alternative for fabricating advanced separators due to its ability to provide tunable porosity, controlled pore distribution, and functional surface layers. These features can significantly enhance device performance by improving ion transport and stability [16].

NC-based separators also exhibit excellent electrolyte wettability and achieve high ionic electrolyte doping levels, meeting the operational requirements of supercapacitor cells under high current densities. Additionally, their hydrophilic nature, combined with strong mechanical, thermal, and chemical stability, has garnered significant attention in recent years for use in advanced energy storage systems [17].

The development costs of nanocellulose (NC)-based separators are significantly lower than those of separators derived from fossil-based materials, such as polyolefins, perfluorinated compounds, and aromatic ionomers. This cost efficiency is an additional advantage of NC-based separators. Compared to commercial polymeric separators—such as microfibrous polypropylene (PP), PP/PE/PP tri-layer composites, and branded separators like Viledon^®^ and Nafion^®^—NC-based membranes offer superior mechanical, thermal, and hydrophilic properties.

Early research by Chun et al. demonstrated the potential of nanofibrillated cellulose (NFC)-based films as replacements for commercial diaphragms [18]. The densely packed cellulose nanofiber (CNF) structure in these films creates a labyrinth-like nanofibrous network. This configuration allows for the rational design of layered or asymmetric porous structures in heterogeneous nano pads. Active chemical components, such as TPY and PVP, incorporated into the separators exhibit synergistic effects, particularly in mitigating adverse reactions caused by Mn^2+^ ions. This enhancement significantly improves high-temperature cycling performance, achieving a capacity retention rate of approximately 80% after 100 cycles—far surpassing the 5% retention rate of traditional PP/PE/PP separators.

In the development of all-solid-state supercapacitors, polymer matrices blended with acids, bases, ionic liquids, or salts can serve dual purposes as both electrolytes and separators. Advancements in solid-state supercapacitors have led to designs using entirely bio-based materials, further supporting the sustainability of NC-based technologies [19].

Bacterial nanocellulose (BNCs) is a novel nanomaterial with unique properties that is produced by different aerobic bacterial species. BNCs are synthesized in two steps: the first is the production of β 1,4-glucan chains, and the second is the crystallization of cellulose. The conversion of glucose to cellulose involves the following four enzymatic steps. First, phosphorylation of glucose by glucokinase to glucose-6-phosphate (G6P); second, isomerization of G6P to glucose-1-phosphate (G1P) by phosphoglucomutase (PGM); third, conversion of G1P to uridine diphosphate glucose (UDP-glucose) by UDP-glucose pyrophosphorylase; and finally, synthesis of cellulose from UDP-glucose by bacterial cellulose synthetase (Bcs) [20].

Various aerobic bacterial species are capable of synthesizing bacterial nanocellulose (BNCs), a nanomaterial with unique properties. The mechanism of BNCs synthesis involves two steps, the first is the production of β 1,4-glucan chains, and the second is the crystallization of cellulose. Four enzymes are involved in the utilization of glucose to cellulose: Glucokinase performs the phosphorylation of glucose to glucose-6-phosphate (G6P); followed by the isomerization of G6P to glucose-1-phosphate (G1P) by phosphoglucomutase (PGM); the third step involves the conversion of G1P to uridine diphosphate glucose (UDP-glucose) by UDP-glucose pyrophosphorylase; and finally cellulose is synthesized from UDP-glucose by bacterial cellulose synthetase (Bcs) [18].

Nanocellulosic materials have different physicochemical properties depending on the methods used for their preparation. Based on these properties, three types of nanocellulosic materials can be distinguished: cellulose nanocrystals (CNC), cellulose nanofibrils (CNF), and bacterial nanocellulose (BC) [21]. In contrast to plant cellulosic materials, bacterial nanocellulose has some advantages, the absence of hemicellulose and lignin, high crystallinity, as well as high crystallinity and water absorption capacity, good mechanical properties, and a large surface area. The fibers of bacterial nanocellulose are about 20–100 nm in diameter. Bacterial nanocellulose is naturally produced by some species of bacteria. One of the first known producers of (nano)cellulose is *Komagataeibacter xylinus* (formerly known as *Gluconacetobacter xylinus*) [22,23]. A sustainable way to produce nanocellulose is through a symbiotic culture of bacteria and yeast (SCOBY). This culture is capable of producing cellulose during the fermentation process in a medium prepared from brewed tea with added sucrose. Kombucha bacterial cellulose (KBC) is produced by acetic acid bacteria in the form of a biofilm that is composed of multiple layers and increases in thickness with increasing fermentation time under suitable environmental conditions. SCOBY consists of acetic acid producing bacteria (Komagataeibacter, Gluconobacter, Acetobacter species) (AAB), lactic acid bacteria (Lactobacillaceae, Lactococcus) (LAB), bifidobacteria, and yeasts (Schizosaccharomyces pombe, Saccharomycodes ludwigii, Kloeckera apiculata, Saccharomyces cerevisiae, Zygosaccharomyces bailii, Torulaspora delbrueckii, Brettanomyces bruxellensis) depending on raw materials, starter cultures, and fermentation temperature [24].

The utilization of bio-based residues is a critical metric for achieving sustainable development goals (SDGs). In alignment with these goals, the circular economy aims to minimize waste and maximize the life cycle of products by repurposing residual materials into valuable resources [25]. Our study exemplifies this approach by upcycling industrial food waste into sustainable nanocellulose. This process not only reduces waste but also adds significant value by converting discarded materials into high-performance nanocellulose products. In our work, we successfully demonstrated a sustainable and environmentally friendly method for producing nanocellulose using food waste as the raw material. The resulting Kombucha bacterial cellulose (KBC) membrane was evaluated for its potential in energy storage applications. Specifically, we conducted performance studies on a supercapacitor cell using the KBC membrane, highlighting its suitability for advanced, eco-friendly energy solutions.

BNC also finds applications in SC, as it can serve as an ideal layered matrix for the incorporation of active two-dimensional (2D) materials. A novel strategy for the incorporation of graphene oxide (GO) sheets into layered BNC during its growth is presented, which prevents their restacking and loss of active area and leads to excellent energy storage performance as well as mechanical flexibility Yuan et al. used BNC as a substrate for deposition of polypyrrole (PPy) and bimetallic hydroxide (NiMn-LDH) by successive layer assembly [26]. The synthesized electrode demonstrated excellent performance in terms of the capacity of 653.1 Cg^−1^ at 1 Ag^−1^. When it was used as a cathode with an Fe_3_O_4_@C/MWCNT anode, it demonstrated an energy density of 29.8 WhKg^−1^ and power density of 299 Wkg^−1^. Song et al. developed a simple, scalable approach to producing hybrid paper from PANI/graphene and CF, which was bridged by BNC as a scaffold. The prepared hybrid paper exhibited superior areal capacitance along with outstanding flexibility. The all-solid-state device fabricated demonstrated an aerial capacitance of 630 mF cm^−2^ and energy density of 2.8 mWh kg^−1^ [27]. To avoid aggregation of CNT and increase the wettability of supercapacitor, bacterial cellulose is used in fiber-based supercapacitor (FSC) where hierarchical core-sheath and porous structure of PPy@CNT-BNC was designed by Yao et al. Fabricated all-solid-state FSC showed an energy density of 8.3 Wh kg^−1^ at a power density of 47.3 Wkg^−1^ along with outstanding capacitance retention and bending ability [28].

Bacterial nanocellulose (BNC) has also been extensively studied as a separator in lithium-ion batteries, with findings indicating enhanced performance for these energy conversion devices [29,30,31].

In the present study, BNC was synthesized through a multi-step process involving the growth of bacterial cultures, isolation of the crude extracellular matrix, chemical treatment/purification, and a specially designed drying method. This approach yielded homogeneous, dense, and thin pristine BNC membranes with thicknesses ranging from 50 to 150 microns. To further enhance the properties of the BNC membranes, a portion underwent surface modification using a high-volatility alkali-alcohol meta-polybenzimidazole (m-PBI) impregnation solution. This modification resulted in hybrid BNC/PBI membranes with improved chemical stability and tailored ion transport properties. The newly prepared BNC membranes were subsequently doped with a 6 M KOH solution to impart high OH^−^ conductivity and were electrochemically tested as a separation membrane in a laboratory-scale supercapacitor cell to improve its capacitance and energy performance, and the results were compared with a supercapacitor cell operating with commercial Viledon^®^ as well as a newly prepared PBI impregnated Viledon^®^ separator.

## 2. Experimental

### 2.1. Materials

Commercial S-26 meta-polybenzimidazole solution, 0.7 dlg^−1^ (PBI Performance Products, Inc., Charlotte, NC, USA), commercial Viledon^®^ separator (700/F), KOH technical grade (Valerus Co., Sofia, Bulgaria), ethanol 96% from local supplier, YP 50 F (Kurary GmbH, Hattersheim am Main, Germany).

### 2.2. Preparation of BNC Membranes

#### 2.2.1. Microbial Synthesis and Isolation of Kombucha Culture BNC

In the present study, the kombucha starter culture was obtained from a commercial drink in Bulgaria. The green tea kombucha used in this study was prepared using the method routinely employed by the company. Briefly, green tea leaves were infused for 30 min in 1.0 L of boiling water with a concentration of 2 g/L. The tea infusion was then filtered into 250 mL flasks containing 150 mL of liquid medium, to which 5% (*w*/*v*) sucrose was added. The mixture was inoculated with 10% (*v*/*v*) of the kombucha beverage starter culture. The kombucha tea was incubated for 21 days at 30 °C with a pH of 6.0, without agitation.

Kombucha cellulose, the jelly-like membrane known as the zooglea biofilm, is formed by symbiotic yeast and bacteria adhering together. The formation of the zoogleal biofilm occurs when bacteria adhere to the surface of the aqueous environment and excrete a polysaccharide matrix that holds the biofilm together [26]. Purification of BNC was carried out using the method described by Sheykhnazari et al. [25]. Briefly, the crude kombucha bacterial nanocellulose sponges collected on day 21 were washed with distilled water. The microbial-grown BNC hydrogel sponges, which had a cylindrical shape (average diameter 10 cm, average height 15 mm), were then boiled in distilled water at 90 °C for 2 h. They were subsequently boiled in a 0.5 M NaOH solution for 15 min at 90 °C to remove residual proteins, polyphenolic compounds, and other impurities. The purified pale white BNC super-hydrogel sponges were then washed several times with distilled water until a neutral pH was achieved.

#### 2.2.2. Drying of Super-Hydrogel BNC Sponges

In order to obtain self-supporting BNC films and to suppress the known strong adhesion of BNC hydrogels to glass and olefin/methacrylate-based plastic substrates, we placed the purified wet BNC patches directly onto a Teflon sheet (20 × 20 cm). The drying process was carried out in a ventilated oven at 60 °C until a constant weight was achieved, which took 3 days. In this manner, dry, robust, and flexible self-supporting BNC films with an average thickness of 80 microns were obtained.

#### 2.2.3. Impregnation of Dry BNC Films with an Alkali–Alcohol PBI Solution

A one-weight percent ethanolic KOH solution of regenerated meta-PBI was used for the impregnation of dry BNC films. The preparation of the ethanolic KOH meta-PBI binder solution was carried out as previously described [32]. Briefly, a 5 × 5 cm dry BNC film was dip-coated in a beaker containing 30 mL of the impregnating PBI solution. After 30 min of contact and a short ultrasound treatment to remove any entrapped air in the BNC fibrous network, the impregnated BNC samples were removed, and the excess PBI solution was drained by gravitational efflux. Gentle capillary suction using filter paper from one of the edges helped remove the remaining solution. The still-wet impregnated BNC/PBI film was then gently dried using a laboratory hot air gun for several seconds until the ethanol had fully evaporated. Next, the impregnated BNC sample was washed with distilled water to remove any residual KOH and then dried in a vacuum oven at 50 °C overnight. The achieved impregnation of BNC was 5 wt.% on a dry BNC base.

#### 2.2.4. Doping of BNC and BNC/PBI Separator Membranes with KOH Electrolyte

Preliminarily weighted square-shaped 5 × 5 cm samples of both pristine BNC and BNC/PBI dry films were immersed in a 6 molar potassium hydroxide (KOH) electrolyte for 6 h. After this, the samples were removed and gently pressed between two pieces of filter paper in order to remove the excess KOH solution from the surface of the swelled membranes. The degree of swelling of the KOH-doped membranes was calculated gravimetrically using the following equation:

(1) $$Degree\;of\;swelling\left(\%\right)=\frac{M_{swell}-{\;M}_{dry}}{M_{dry}}\times 100$$

where *M_swell_* is the mass of the KOH doped membranes and *M_dry_* is their dry mass accordingly.

The same procedure was applied to the reference Viledon^®^ and PBI-impregnated Viledon^®^ membranes.

#### 2.2.5. Physicochemical Characterization of BNC and BNC/PBI Membrane

The morphology of the dry BNC films was visualized and evaluated using a Leica DMLP Optical Microscope and Wide-Angle X-ray Diffraction (WAXD). WAXD scans were conducted with a Bruker D8 Advance ECO diffractometer (Bruker, Billerica, MA, USA), operating at 40 kV and 25 mA in Bragg–Brentano geometry. The diffractometer used Ni-filtered Cu Kα radiation and a LynxEye-XE detector, covering the 2θ range from 5° to 50°, with a scanning rate of 0.02°·s^−1^. The crystallinity index (*Cr*.*I*) of BNC was calculated from XRD measurements using Segal’s method, with the formula:

(2) $$Cr.I=\left(\frac{I_{200}-I_{am}}{I_{200}}\right)\times 100$$

where *I*_200_ and *I_am_* represent the maximum diffraction intensities at 2θ = 22.7° and 18°, respectively.

Thermal analysis was performed using a Perkin Elmer Pyris thermogravimetric analyzer (TGA), with the sample heated from 40 °C to 800 °C at a rate of 15 °C min^−1^ under continuous nitrogen purging. Fourier-transform infrared (FTIR) spectroscopy was carried out with a Thermo Fisher Scientific Nicolet iS50 FTIR spectrometer. The ATR spectra were averaged over 50 scans in the range of 4000 to 500 cm^−1^ and recorded at a resolution of 4 cm^−1^. All measurements were performed at room temperature.

The BET analysis was conducted on a NOVAtouch–Quantachrome instrument (USA), which measures adsorbed or desorbed gas volumes at a relative pressure of less than unity. The data obtained after computer processing are presented as adsorption isotherms, from which specific surface area, pore volume, pore size of solid, and powder samples are calculated.

### 2.3. Preparation of the Carbon Electrodes

The electrode mass consisted of 80 wt.% YP 50 F (Kurary GmbH), 10 wt.% acetylene black, and 10 wt.% polytetrafluoroethylene (PTFE). This active mass was applied onto nickel foam with a surface area of 0.64 cm², followed by drying at 120 °C under vacuum and pressing at a pressure of 2 MPa. The resulting electrodes were incorporated into a two-electrode Swagelok coin cell configuration. BNC and BNC/PBI membranes were used as separators, with 6 M KOH as the electrolyte. For comparison, a Viledon^®^ separator was used as the reference for alkaline media.

### 2.4. Electrochemical Characterization

Electrochemical measurements of supercapacitors were conducted using cyclic voltammetry (CV) across various scan rates, ranging from 10 to 50 mV s^−1^, within a voltage window of 0.05–1.2 V. This was carried out utilizing a Multi PalmSens system (Model 4, Netherlands). Electrochemical impedance spectroscopy (EIS) measurements were also performed using the same equipment, covering frequencies from 10 MHz to 1 mHz. Galvanostatic charge–discharge measurements were executed employing an Arbin Instrument System LBT-21084, spanning from 0.0 to 1.2 V. The test protocol involved maintaining a constant current load ranging from 30 to 1000 mAg^−1^ for a 30 number of cycles per step. For long-term cycling tests, the cells underwent continuous cycling at a current rate of 240 mAg^−1^ for 10,000 charge–discharge cycles. The specific capacitance, *C_s_* (Fg^−1^), derived from cyclic voltammetry, was calculated as follows:

(3) $$C_{s}=\frac{4I\frac{dV}{dt}}{m}$$

where *I* is the current, *dV*/*dt* is the voltage scan rate, and *m* is the mass of the active carbon material.

The following equation was used to calculate the specific capacitance (Fg^−1^) from the charge/discharge curves:

(4) $$C=\frac{4I\Delta t}{mdV}$$

where *I* (A), *Δt* (s), *m* (g), and *ΔV* (V) indicate the discharge current, discharge time, mass of the active material, and voltage window, respectively.

## 3. Results

### 3.1. BNC Based Membranes Preparation and Their Alkali Doping

**Figure 1:**
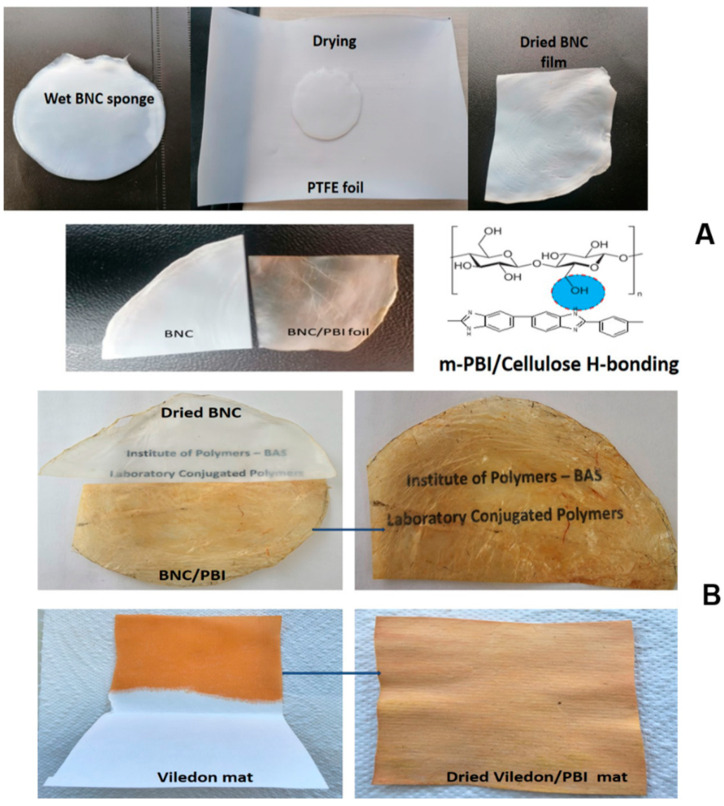
Digital images representing different stages of the crude microbial BNC membrane substrate drying/impregnation process for BNC and BNC/PBI separator films prior to KOH doping (**A**). Pictures of the prepared semi-transparent dry BNC and BNC/PBI films and the commercial Viledon^®^ microfibrous mat impregnated with alkali–alcohol PBI solution (**B**).

In the present study, we used microbially grown super-hydrogel substrates composed of filamentous bacterial nanocellulose. After several purification and drying steps, these substrates resulted in mechanically robust, strong, and flexible pristine dry BNC films with an average thickness of 80 microns. A particular challenge was finding an appropriate drying procedure to effectively dry the super-hydrogel nanofibrous cellulose sponges (which contained 1100 wt.% water on a dry cellulose base) in order to obtain thin, self-supporting BNC films suitable for subsequent alkaline electrolyte doping. In another approach, we employed a volatile ethanolic KOH solution of commercial meta-PBI with a 1 wt.% polymer concentration to efficiently impregnate the BNC, forming polymer-polymer complexes. This process bonds the cellulose surface’s -OH groups with the imidazole repeat units of PBI, facilitated by hydrogen bonding and hydrophobic interactions, as depicted in Figure 1A.

Both the pristine dry BNC and PBI-impregnated BNC films were semi-transparent, with the PBI-impregnated samples showing an improvement in translucency. For comparison of properties, commercially available Viledon^®^ microfibrous polypropylene nonwoven separators, commonly used in batteries and supercapacitors, as well as laboratory-prepared PBI-impregnated Viledon^®^ mats, were employed (Figure 1B).

The degree of PBI impregnation of both BNC and Viledon^®^ mats was approximately 5 wt.%. Although recent research has focused on the use of BNC-based membranes as separators in supercapacitor cells, with various approaches for surface modification using different polymers, inorganic filler composites, and dopant electrolytes, we were unable to find data on BNC impregnation with polybenzimidazoles or the use of simple alkali KOH electrolyte as an OH- conductivity dopant. It is well-known that filamentous cellulosic materials, when in continuous contact with concentrated alkali metal hydroxide solutions, undergo a swelling process known as mercerization. The cellulosic hemi-acetal residue bridges of the polymer’s main chain are stable in alkaline media at ambient temperature. Non-flammable and chemically robust PBI is also known to form solid polymer electrolyte materials when doped with alkali bases (e.g., LiOH, NaOH, KOH) [33].

The equilibrium degree of swelling of dry BNC films doped with a concentrated 6 M KOH solution reached 195%, which corresponds to 5.5 moles of KOH dopant per repeat cellulosic unit. For the PBI-impregnated BNC, slight hydrophilization of the hybrid membrane was observed, with a degree of swelling (DS) of 170% and 4.8 moles of KOH dopant per repeat cellulosic unit. The swelling of both BNC and BNC/PBI membranes doped with 6 M KOH can be considered moderate. A control experiment with regenerated cellulose film (Cellophane), which had a similar thickness, showed a much higher degree of swelling—450% after 6 h of contact with a 6 M KOH solution. However, over-swelling of this cellulosic material was observed overnight, resulting in a loss of mechanical robustness. For comparison, commercial microfibrous polypropylene-based Viledon^®^ mats were tested as supercapacitor cell separators. The measured DS for the pristine Viledon^®^ mat was 370%, while impregnation with PBI resulted in a significant reduction in DS to 205%.

### 3.2. Characterization of BNC and KOH Doped Membranes

Crystalline cellulose exhibits various forms based on its source and the processes it undergoes. It is widely recognized that various crystalline polymorphisms (I, II, III, IV) of cellulose exist [34]. Type I is a native cellulose found in most plant cell walls and structures; type II is textile cellulose that can be obtained by processing with strong alkalis during heat treatment. Cellulose types III and IV are less widely found and occur in combination with types I and II depending on the degree of treatment. The bacterial cellulose is composed of cellulose type I, and it contains two regions—crystalline and amorphous. The crystalline region exists in two different polymorphs—I_α_ and I_β_. The I_α_ structure is represented by the triclinic system with one chain in a unit cell, while the I_β_ structure is monoclinic with two chains per unit cell. Bacterial cellulose was repeatedly reported to possess a considerable amount of the I_α_ coexisting with the I_β_.

**Figure 2:**
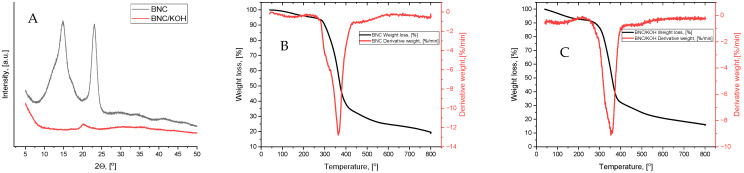
WAXD diffraction patterns (**A**) and TGA curves (**B**) of the dry neat BNC film and dry BNC film after KOH treatment (**C**).

WAXD patterns of the dry neat BNC film and dry BNC film after KOH treatment are shown in Figure 2A. In previous studies, the bacterial cellulose was reported to possess a cellulose type I structure represented by two intense peaks at 14.7 and 22.7°, and one smaller, less intense one at 22.7° 2θ, corresponding to (110), (110), and (200) planes, respectively. The diffraction peak at 14.7° is characteristic of cellulose I_α_, and that at 22.7° *2θ* of cellulose I_β_, the calculated Cr.I is almost 60%. In contrast, the WAXD pattern of dry BNC film after KOH treatment is completely different. It is well known that the strong alkali solution causes changes to the crystal structure and transition from cellulose type I to cellulose type II. After KOH treatment of neat BNC film, a complete transition from cellulose type I to type II is observed. Cellulose type II is represented by a small diffraction peak at 19.9° 2θ in (110) plane direction [35].

The TG/DTG curves of the dry neat BNC film and dry BNC film after KOH treatment are shown in Figure 2B,C. In a low temperature <100 °C range, an initial small weight loss is observed due to the evaporation of absorbed water. The pronounced degradations of the neat BNC film started at approximately 285 °C, while the degradation of BNC/KOH film started at 275 °C. The temperatures of the maximum degradations of BNC and BNC/KOH samples were 355 °C and 365 °C, respectively, which is relevant to those reported in the literature [36]. In general, the BNC film treated with KOH exhibited a lower degradation temperature due to a larger number of free ends of chains in BNC/KOH caused by the alkali treatment. Regardless of the polymorph that has changed and cellulose mercerization, both samples had a similar thermal degradation trend.

**Figure 3:**
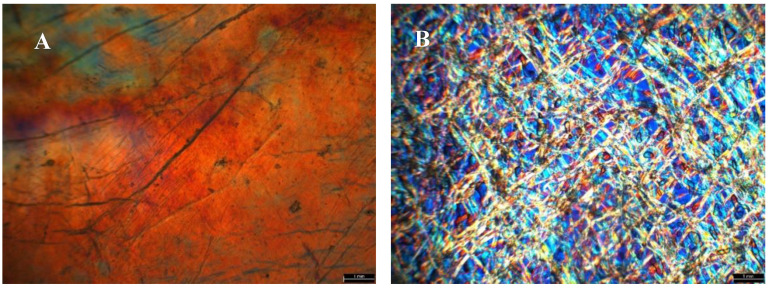
POM images of BNC before (**A**) and after (**B**) KOH treatment.

Polarized optical microscopy (POM) imaging was used to qualitatively study the birefringence of the cellulose samples (Figure 3). Samples were placed between two cross-polarizers in an optical microscope (Leica DMLP Optical Microscope) and interference color angles were determined at ±45°. As can be observed (Figure 3), the treatment of the BNC dry film with 6 M KOH solution exposes the self-formation of a highly swollen anisotropic microfibrillar network on the top of the KOH doped BNC membrane within the dense hydrogel bottom layer.

### 3.3. FT-IR Analysis of BNC Membranes

**Figure 4:**
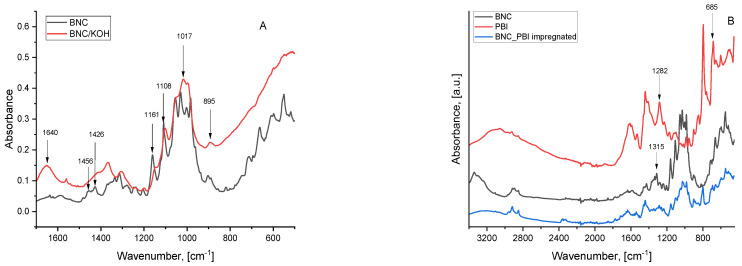
ATR-FTIR spectra of dry neat BNC film and dry BNC film after KOH treatment (**A**) and BNC impregnated with PBI (**B**).

To characterize the BNC membranes, FTIR-ATR analysis was employed. The ATR-FTIR spectra of both the dry neat BNC film and the dry BNC film after KOH treatment are presented in Figure 4.

In Figure 4A, the spectrum of the original membrane and after doping with KOH is presented. The analysis reveals a spectrum containing characteristic bands related to cellulose type I. The peak at 3344 cm^−1^ is indicative of -OH stretching vibrations in cellulose type I. The characteristic bands for cellulose type I are also observed at 2893 cm^−1^, corresponding to CH stretching of CH_2_ groups, and the asymmetric CH_2_ stretching at 2853 cm^−1^. The peaks in the region of 1650–1640 cm^−1^ are attributed to the carboxyl functional group (C=O) and H-O-H bending vibrations due to water adsorption. The peak at 1426 cm^−1^ corresponds to CH_2_-symmetric bending, while the distinct peak at 1161 cm^−1^ indicates C-O-C asymmetric stretching at the β-glycosidic bond [37,38].

After doping with KOH, significant changes in the BNC spectrum are observed in the region of 1650–850 cm^−1^. Alkaline treatment affects the structure of cellulose. The peak corresponding to the glucopyranose ring at 1108 cm^−1^ associated with β-glycosidic bonds increases, indicating partial amorphization of the BNC. Similarly to the results published in a previous study [39], the peaks corresponding to atoms involved in hydrogen bond formation—in the region between 1056 cm^−1^ and 985 cm^−1^—lose their sharpness and become broadened. The same effect is observed in the region from 1456 cm^−1^ to 1426 cm^−1^, where the two peaks corresponding to bending vibrations of the C-O-H groups smooth out. The results indicate that after doping the membrane with KOH, the hydrogen bonding network in the initial BNC is partially disrupted, leading to loosening and destabilization of the structure. After treatment with the alkaline solution, the distinct peak at 1640 cm^−1^, corresponding to O-H bending vibrations, along with the increased intensity of the signal at 895 cm^−1^, associated with the stretching of β-glycosidic bonds typical of amorphous regions in cellulose, further confirms the partial amorphization of the structure and its enhanced ability to absorb and retain water, particularly in these amorphous areas.

In Figure 4B, the FTIR spectrum of BNC after impregnation with an alkali–alcohol meta-polybenzimidazole (m-PBI) solution is shown, alongside the spectra of pure m-PBI and neat BNC as references. The spectrum of the BNC impregnated with m-PBI displays characteristic peaks of both polymers, though some notable differences are observed. In the combined spectrum, the peaks at 1315 cm^−1^ and 1345 cm^−1^, typically associated with bacterial nanocellulose, are no longer present. These peaks correspond to C-H deformations in the cellulose molecule, particularly in the methylene groups (CH_2_) of the glucose units, and to O-H bending vibrations and C-H vibrations in the glucose rings of cellulose, respectively. A plausible explanation for this observation is that the formation of hydrogen bonds between BNC and m-PBI alters the vibrational modes, leading to a smoothing effect that weakens or eliminates the characteristic peaks of m-PBI. Another notable change observed in the combined spectrum is the decrease in the intensity of the peaks at 685 cm^−1^ and 1282 cm^−1^ compared to the spectrum of pure m-PBI [40]. This change is likely due to the interaction with bacterial nanocellulose and the formation of hydrogen bonds, which alter the electron density of the C-N bonds. Consequently, this leads to a weakening of the associated vibrations and, respectively, a reduction in the intensity of the peaks. The appearance of one new peak at 1260 cm^−1^ corresponding to NH2 deformation proves the presence of a hydrogen bonding net.

### 3.4. BET Surface Area and Pore Size Analysis

The adsorption isotherms of nanocellulose membranes, designated BNC-pure and BNC/PBI, were measured by physisorption of nitrogen gas. The isotherms are presented in the figure.

**Figure 5:**
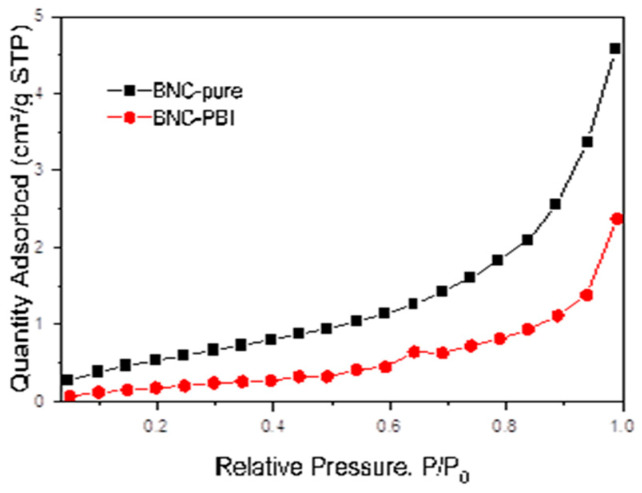
Nitrogen adsorption isotherms of BNC-pure and BNC/PBI.

The measured adsorption isotherms for BNC-pure and BNC-PBI (Figure 5) are closest to Type II according to the IUPAC classification. Isotherms of this type are obtained from the physical adsorption of most gasses on non-porous or macroporous adsorbents. The shape is a result of the unconfined monolayer–multilayer adsorption up to high p/p_0_.

The stage at which the monolayer coverage is complete and multilayer adsorption begins is marked by an inflection point of the isotherm (point B). In this case, point B is difficult to distinguish, which is an indication of a significant overlap between the monolayer and the beginning of multilayer adsorption. The thickness of the adsorbed multilayer usually increases without limit when p/p_0_ = 1 [41].

From the adsorption isotherms, the structural characteristics were determined and summarized in the Table 1.

The Surface Area was determined by the BET method (Brunauer, Emmett, Teller). Multipoint BET was determined at relative pressure in the range p/p_0_ = 0.1–0.3. The Pore Volume is calculated at a relative pressure of measurement close to 1 (p/p_0_ = 0.99). The Average Pore Diameter is calculated assuming that the pores have a cylindrical geometry, at p/p_0_ = 0.99.

The measured structural characteristics of the nanocellulose membranes BNC-pure and BNC-PBI show different surfaces, with BNC-PBI having a lower specific surface area of 0.92 m^2^/g and a smaller mesopore volume of 0.0037 cm^3^g^−1^, compared to BNC-pure with a surface area of 2.3 m^2^g^−1^ and a mesopore volume of 0.0071 cm^3^g^−1^.

It can be concluded that these nanocellulose membranes have relatively low surfaces, do not have a developed pore volume for pores with a diameter below 160–190 nm (D), and have a similar average pore diameter of 12–16 nm.

### 3.5. Electrochemical Characterization

The supercapacitor cells underwent testing under uniform experimental conditions, allowing for comparisons with BNC and BNC/PBI separators. Additionally, investigations were carried out using the traditional Viledon^®^ separator designed for alkaline electrolytes and also Viledon^®^/PBI. To observe changes in the electrode, cyclic voltammetry (CV), and electrochemical impedance spectroscopy (EIS) curves were recorded both before and after a long-term cycling test (10,000 cycles at 240 mAg^−1^).

**Figure 6:**
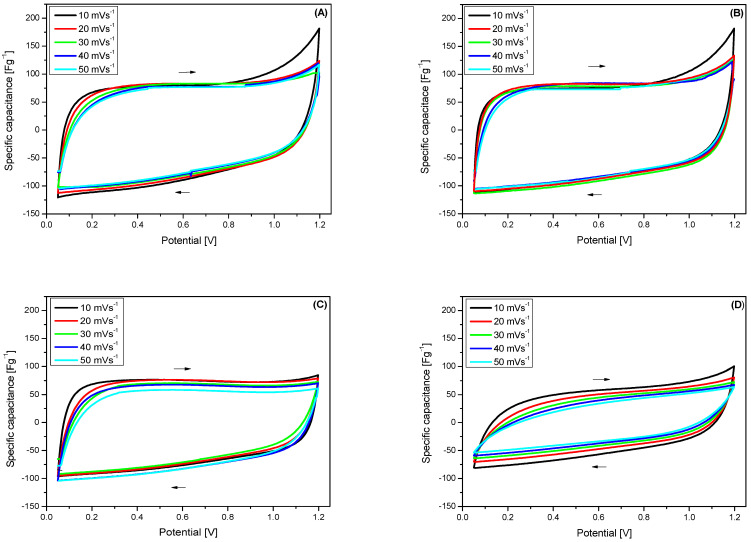
CV curves of symmetric supercapacitors with different membranes and at different scan rates: (**A**) BNC, (**B**) BNC/PBI, (**C**) Viledon^®^, (**D**) Viledon^®^/PBI.

Figure 6 shows the CV voltammograms for SCs with BNC (Figure 6A), BNC/PBI (Figure 6B), Viledon^®^ (Figure 6C), and Viledon /PBI (Figure 6D), before galvanostatic charge/discharge cycling. Overall, all voltammograms using BNC, BNC/PBI, and Viledon^®^ display typical characteristics of symmetric supercapacitor systems with nearly rectangular shapes, which remain consistent as the scan rate increases from 10 to 50 mV s^−1^. The rectangular shape of the voltammograms is retained even at higher scan rates, indicating excellent capacitive behavior of the devices [42]. However, the CV profile of the SC with the Viledon^®^/PBI membrane is non-rectangular, likely due to the higher resistance of this membrane. It appears that impregnation with PBI does not favorably affect the overall ionic conductivity.

**Figure 7:**
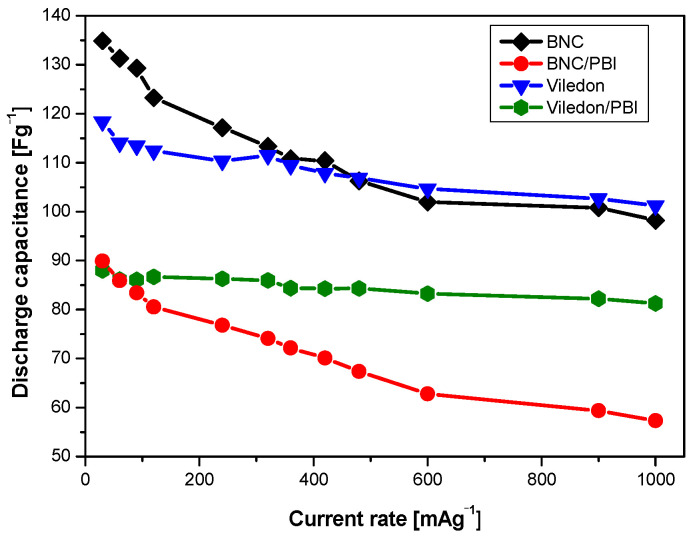
Discharge capacitance vs. current rate of symmetric supercapacitors with different membranes.

**Figure 8:**
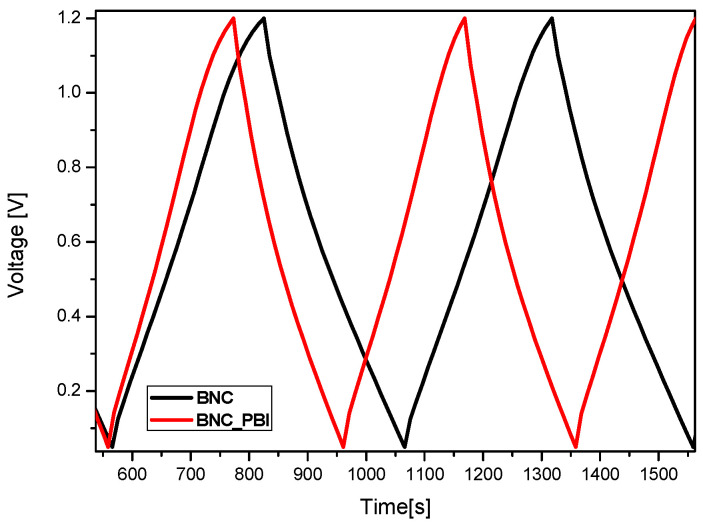
Galvanostatic charge/discharge curves of symmetric supercapacitors with BNC and BNC/PBI membranes at 240 mAg^−1^.

**Figure 9:**
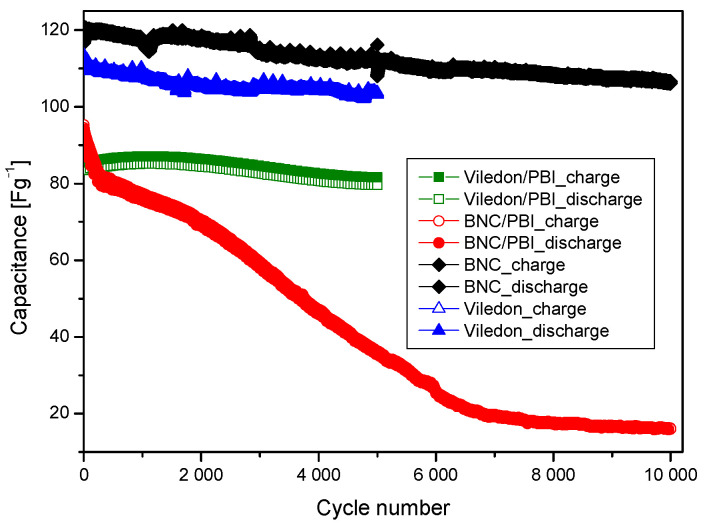
Long-term test of symmetric supercapacitors with various membranes.

The results of the galvanostatic charge/discharge tests are shown in Figure 7, Figure 8 and Figure 9. Figure 7 presents the specific discharge capacitance as a function of the discharge current rate for symmetric supercapacitors with different membrane types (BNC, BNC/PBI, Viledon^®^, Viledon^®^/PBI). It was observed that the specific capacitance decreases as the current rate increases, likely due to enhanced diffusion limitations within the deeper pores of the YP-50F activated carbon [43].

An analysis of the cycling behavior indicates that symmetric supercapacitor cells exhibit good reproducibility in discharge capacitance. Among the configurations tested, the symmetric supercapacitor with the BNC separator demonstrated the highest discharge capacitance. In line with the observations in Figure 6, the Viledon^®^/PBI configuration proved to be unstable, displaying a significant capacitance drop at higher current loads.

The charge/discharge curves of symmetrical supercapacitors with BNC and BNC/PBI membranes measured at 240 mA g^−1^ are shown in Figure 8. It can be observed that the supercapacitors exhibit stable electrochemical properties in the electrolyte, with the capacitor voltage changing almost linearly in time during both charging and discharging, indicating good capacitive behavior. It should also be noted that in alkaline media, the pseudocapacitive reactions depend on the carbonyl functional groups present on the carbon materials [44]. Although carbon YP-50F is basic in nature, it contains acidic functional groups, such as phenolic, carbonyl, and carboxyl groups [45], which contribute to its capacitive properties, which is in good agreement with the voltage profiles shown in Figure 8. The observed voltage drop values during discharge are low, with no significant difference between the two membranes, ranging from 0.11 to 0.12 V.

Figure 9 illustrates the capacitance retention of the electrodes over long-term cycling. The supercapacitor with a BNC membrane as the separator shows the highest capacitance stability among the cells studied, maintaining 88% of its initial capacitance after 10,000 cycles. This stability can be attributed to the inherent properties of the BNC membrane. In contrast, the adverse effect of PBI impregnation is evident in the sharp decline in capacitance of the symmetric supercapacitors with the BNC/PBI separator, which exhibits two pronounced drops after the 300th and 2000th cycles, ultimately retaining only 17% of its initial capacitance. Additionally, the BNC-based supercapacitors demonstrate high current efficiency, remaining above 99.5% throughout the entire cycling test.

**Figure 10:**
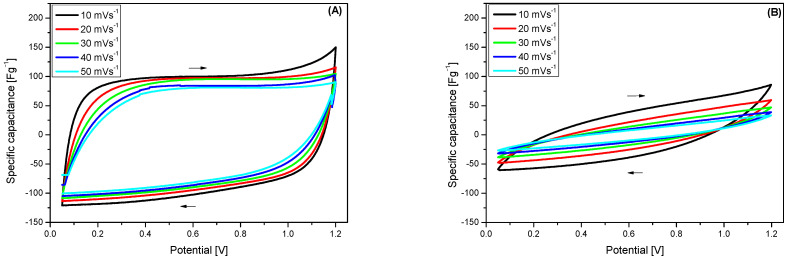
CV curves of symmetric supercapacitors with BNC- membranes at different scan rates after 10,000 charge/discharge cycles: (**A**) BNC, (**B**) BNC/PBI.

The excellent capacitive behavior of the BNC membrane supercapacitor is also demonstrated in Figure 10, which shows the CV curves of symmetric supercapacitors with BNC and BNC + PBI membranes at different scan rates after 10,000 charge/discharge cycles. As can be seen from the figure, the symmetric supercapacitor with BNC membrane maintains the orthogonal shape of the voltammograms at all scan rates, indicating outstanding capacitive characteristics even after long cycles. In contrast, supercapacitors with BNC/PBI membranes show a distortion in the voltammogram shape, which is likely due to the PBI doping in the BNC structure. This deformation implies reduced capacitive efficiency as PBI-cellulose intermolecular H-bonding interactions and the physical PBI layer formation within cellulosic nanofibrils alters both the conductive and structural properties of the membrane in a more or less negative aspect for impregnated membrane samples, as is visible from the performed CV analysis.

**Figure 11:**
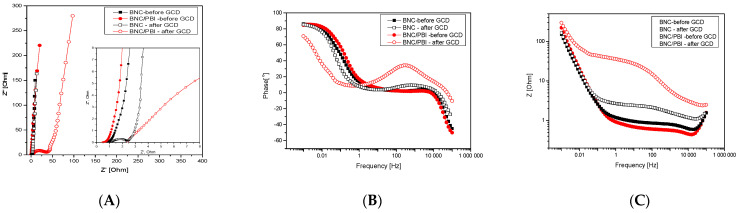
EIS of studied SC before and after 10,000 GCD: (**A**) Nyquist plots (full range (**left**) and zoomed (**right**), (**B**,**C**) Theta as a function of the frequency and (**C**) Bode plots.

Electrochemical impedance spectroscopy (EIS) was conducted to analyze the impedance characteristics of BNC and BNC/PBI membranes, measured both at the beginning of the tests and after 10,000 cycles (Figure 11).

As could be observed from the Nyquist plot characteristics, the freshly doped pure BNC and PBI impregnated BNC separators in a real supercapacitor cell operational mode showed similar ohmic resistance characteristics with slightly better performance of the PBI impregnated membrane. The curves clearly reflect a variation in the cell resistance and capacitive behavior of the cells. The semicircle at the high-mid frequency and the vertical line in the low-frequency region are a common concept in the double-layer formation and ion diffusion kinetics at electrodes. A noticeable variation in the plots and change in the Z’ axis at the low-frequency tail is observed. This part of the plot is responsible for the ion interaction with pores, which can be significantly affected by the presence of a PBI polymer.

The interception point on the Z’ axis at the high-frequency region is solution resistance (R_s_), which is from the electrode–electrolyte interaction and electrical contact. The R_s_ values for BNC and BNC/PBI before GCD were 0.48 and 0.62 Ω, respectively. The interception of the semicircle ends with the Z’ axis and is the charge transfer resistance (R_ct_) of the cell. The R_ct_ values for BNC and BNC/PBI before GCD measured were 0.13 and 0.25 Ω, respectively. In general, small R_s_ and R_ct_ are preferred for an excellent supercapacitor system. The impedance spectra of BNC and BNC/PBI membranes after 10,000 cycles show different trends. The R_ct_ value of BNC/PBI membrane increased from 0.25 Ω to 34.8 Ω indicating deterioration of the properties, while the values for the BNC membrane are very similar.

This is consistent with the BET analysis results, which reveal a slight increase in the average pore diameter for the PBI-impregnated BNC membrane (19 nm). The larger pore size provides additional “space” for the free, chemically unbound electrolyte KOH in the membrane volume, despite the lower total electrolyte content (both physically and chemically bound), as determined from the gravimetric swelling ratio (DS) values of the membranes.

## 4. Conclusions

Novel concentrated KOH electrolyte doped bio-based bacterial nanocellulose and meta-polybenzimidazole impregnated nanocellulose membranes were prepared and tested as a separator in a laboratory-scale symmetric supercapacitor cell. The supercapacitor using pristine BNC membranes demonstrated high specific capacitance and excellent cycling stability, maintaining performance over 10,000 charge/discharge cycles. In contrast, the hybrid BNC/PBI membranes showed poorer performance, possibly due to a reduced pore size/specific surface and impaired ion transport during prolonged cycling. The overall performance of the newly tested BNC based alkali-doped separator were compared with a commercial Viledon^®^ and PBI impregnated Viledon^®^ separator, which showed similar cycling stability characteristics, especially for the pristine separator materials. These findings highlight the potential of BNC/KOH doped solid polymer electrolyte membranes for use in supercapacitor devices.

## Figures and Tables

**Table 1:** Structural parameters of BNC-pure and BNC/PBI.

Characteristics	BNC-Pure	BNC-PBI
Surface Area (multi-point BET), m^2^g^−1^	2.3	0.92
Total Pore Volume, cm^3^g^−1^	0.0071 for pores smaller than 164 nm (D)	0.0037 for pores smaller than 194 nm (D)
Average Pore Diameter, (4 V/S), nm	12.3	16.0

## Data Availability

Data are contained within the article.
